# On the Perceptual Subprocess of Absolute Pitch

**DOI:** 10.3389/fnins.2017.00557

**Published:** 2017-10-06

**Authors:** Seung-Goo Kim, Thomas R. Knösche

**Affiliations:** Research Group for MEG and EEG—Cortical Networks and Cognitive Functions, Max Planck Institute for Human Cognitive and Brain Sciences, Leipzig, Germany

**Keywords:** absolute pitch, pitch chroma, ventral auditory pathway, auditory cortex, pitch perception

## Abstract

Absolute pitch (AP) is the rare ability of musicians to identify the pitch of tonal sound without external reference. While there have been behavioral and neuroimaging studies on the characteristics of AP, how the AP is implemented in human brains remains largely unknown. AP can be viewed as comprising of two subprocesses: perceptual (processing auditory input to extract a pitch chroma) and associative (linking an auditory representation of pitch chroma with a verbal/non-verbal label). In this review, we focus on the nature of the perceptual subprocess of AP. Two different models on how the perceptual subprocess works have been proposed: either via absolute pitch categorization (APC) or based on absolute pitch memory (APM). A major distinction between the two views is that whether the AP uses unique auditory processing (i.e., APC) that exists only in musicians with AP or it is rooted in a common phenomenon (i.e., APM), only with heightened efficiency. We review relevant behavioral and neuroimaging evidence that supports each notion. Lastly, we list open questions and potential ideas to address them.

## Absolute pitch

Absolute pitch (AP) is often defined as “the ability to identify the pitch of isolated tones using musical pitch labels or to produce the pitch of any tones designated by note names without comparing to any reference pitch” (Miyazaki, 2004), which is believed to be acquired by predisposition (neural resources) and musical training during a critical period in early childhood (Zatorre, 2003). Unlike a common impression due to historically famous musicians who had AP, this ability is not necessarily beneficial in musical professions—“more akin to a party trick than a useful skill,” as stated by Van Hedger et al. (2015a)—except for some cases, such as musical composition, conducting, or group-wise improvisation in Jazz. For musical performance in non-standard tunings, such as “Baroque pitch” (reference pitch of 415 Hz, unlike the modern standard of 440 Hz), having AP could even be a disadvantage. Correlation between AP and general musical ability may be found sometimes. But it could be due to an early commencement of formal musical training that influences both AP and musical ability (Miyazaki, 2004).

**Figure 1:**
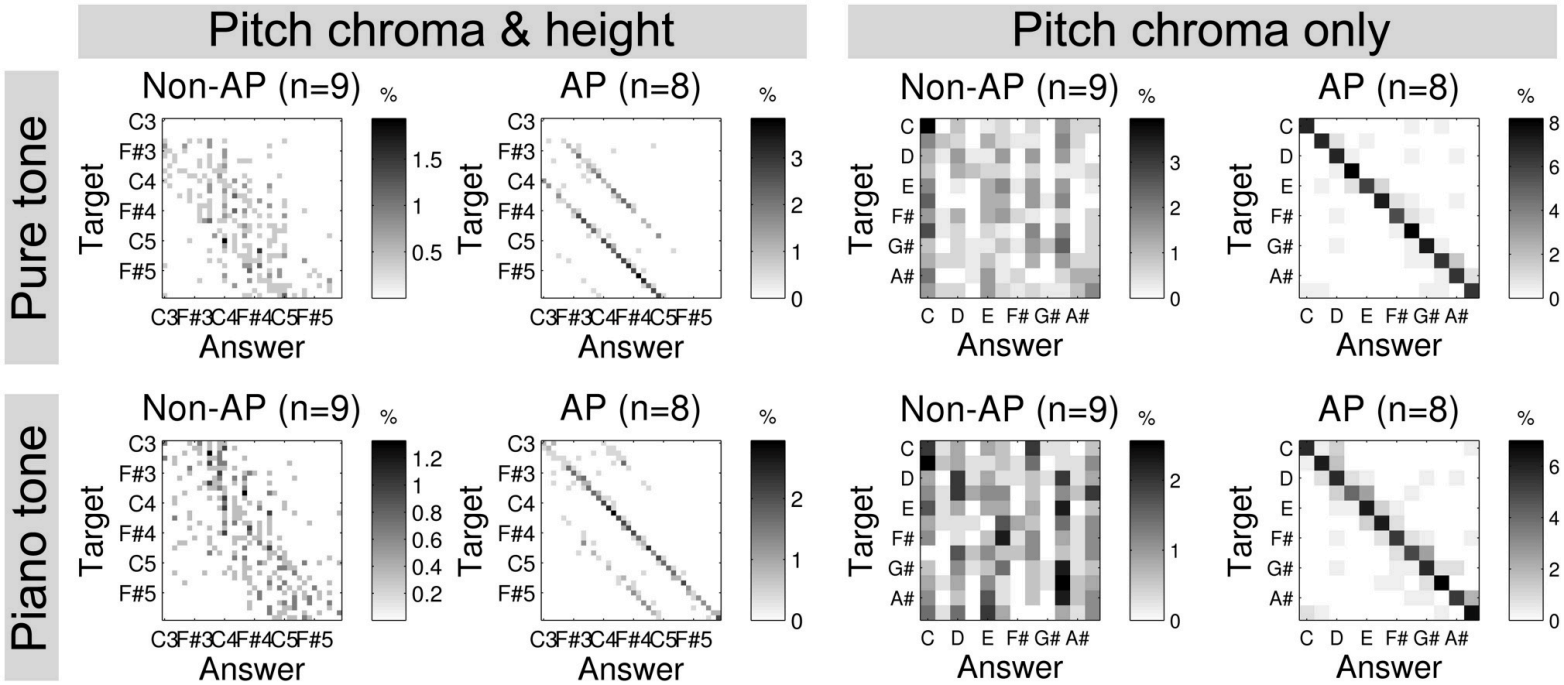
Confusion matrices of an absolute pitch test using sine tones **(top)** and piano tones **(bottom)** by musicians without AP (non-AP) and with AP. Reproduced from Kim and Knösche (2016).

Interestingly, it has been long known (Bachem, 1955) and consistently confirmed in recent behavioral studies (Miyazaki, 1988; Takeuchi and Hulse, 1993; Deutsch and Henthorn, 2004; Deutsch, 2013) that some musicians with AP, who can correctly and rapidly name the pitch chroma of a given tone, make frequent mistakes in pitch height^1^. In an example given in Figure 1, musicians with AP showed frequent octave errors but very accurate pitch chroma recognition. In contrast, musicians without AP reasonably recognized pitch height, but not pitch chroma. This suggests that AP actually consist in the ability to categorize pitch chroma. Importantly, this implies that musicians with AP do not recognize tones by frequency (or periodicity). This is in line with the perceived similarity between tones spaced by octaves (“octave equivalence”) present in the general population, presumably due to phase-locked synchronization across auditory neurons that detect periodicities spanning octaves. Indeed, it was found that a similar neural population was engaged when listening to complex tones spaced by one octave (Briley et al., 2012). More importantly, however, it is essential that AP musicians categorize a pitch into an arbitrary, discrete, and cultural representation (i.e., pitch chroma), which will be further discussed below.

While a number of neuroimaging studies reported the possible involvement of several brain regions (Schlaug et al., 1995; Keenan et al., 2001; Ohnishi et al., 2001; Itoh et al., 2005; Bermudez et al., 2009; Oechslin et al., 2009; Wilson et al., 2009; Loui et al., 2011; Jäncke et al., 2012; Dohn et al., 2014; Elmer et al., 2015), the neural mechanisms of AP remain unclear. Novel studies provide behavioral and neuroimaging evidence (Van Hedger et al., 2013, 2015a,b, 2016; Kim and Knösche, 2016, 2017), questioning previously assumed characteristics of AP behaviors and underlying neural structures and functions. Here, we review the current state of research focusing on the “perceptual subprocess of AP” and discuss its possible neural correlates. Additionally, we list open questions with some ideas as to how to address them.

## Nature of subprocesses of absolute pitch

Because AP can be observed by naming or producing a given pitch, it has been conceptualized as a serialprocess comprising perceptual (i.e., processing a given auditory input to extract pitch chroma; presumably processed in temporal lobes) and associative (i.e., linking an extracted pitch chroma with a verbal/non-verbal label; presumably processed in frontal lobes) subprocesses (Ward and Burns, 1982; Levitin and Rogers, 2005). While it is commonly accepted that the outcome of the perceptual subprocess is a representation of pitch chroma, there have been different views on which operations are done to achieve it.

In one view, “AP consists of ‘pitch memory,’ which is widespread in the population, and ‘pitch labeling,’ which is possessed exclusively by persons with AP” (Levitin and Rogers, 2005). In other words, the perceptual subprocess in musicians with AP is not different from that in equally trained musicians without AP whereas the associative subprocess is different. In line with this, a PET study (Zatorre et al., 1998) found strong activity in the left dorsolateral prefrontal cortex (DLPFC), which is known to be involved in recognition based on short-term and long-term memory, in AP musicians during passive listening and was interpreted as an indication of the associative AP subprocess. The notion that the perceptual subprocess is not unique in musicians with AP has been further corroborated by studies failing to find functional or structural differences in the temporal lobes (Bermudez et al., 2009; Elmer et al., 2013).

Another view is based on the longstanding belief that the categorization of pitch chroma is done “in the same way they categorize letters, words, or common objects” (Siegel, 1974). This view refers to absolute pitch categorization (APC) for the perceptual subprocess of the AP, which assigns a pitch to one of the chromatic categories. While the precise way this categorization works is still moot, a number of MRI studies reported structural and functional features related to AP in the superior areas of the temporal cortices (i.e., the supratemporal planes), which process primary and non-primary auditory information, such as smaller area of the right planum temporale (PT) (Schlaug et al., 1995; Keenan et al., 2001; Wilson et al., 2009), greater cortical thickness in many regions in the superior temporal gyri (STGs) (Dohn et al., 2015), larger volume of the right Heschl's gyrus (HG) (Wengenroth et al., 2014), greater cortical myelination in the right planum polare (PP) (Kim and Knösche, 2016), higher activation in the left PT (Ohnishi et al., 2001), and a negative ERP at an early latency from an electrode over the left posterior temporal lobe (Itoh et al., 2005). This line of evidence strongly suggests that the perceptual subprocess of AP is different from the auditory processing in non-AP population.

The abovementioned conceptual views contrast with each other on whether the perceptual subprocess of AP uses a mechanism that is present in all humans to some extent (i.e., APM) or it is implemented in a unique way that only exists in musicians with AP (i.e., APC). We review relevant empirical evidence to weigh the plausibility of APM and APC being the essence of the perceptual subprocess as follows.

## Absolute pitch memory vs. absolute pitch categorization

For a number of reasons, we cautiously suggest that APM-based comparison may not be the major mechanism underlying the perceptual subprocess of AP. Also, we suggest that auditory processing in highly trained musicians with AP is different from that in equally trained musicians without AP. The main issues are: (1) whether the accuracy of APM is comparable with that of APC, (2) whether APM can be used for pitch chroma categorization, and (3) whether APM is aligned with standard tuning like APC.

Firstly, the existence of APM in the general population due to extensive and long-lasting exposure seems undeniable (Levitin, 1994; Smith and Schmuckler, 2008; Ben-Haim et al., 2014; Van Hedger et al., 2016), although the observed accuracy is usually not very high. For instance, in a singing task of self-selected familiar songs (Levitin, 1994), the mean absolute error (computed from the reported histogram) was around 2 semitones, while the expected mean absolute error (disregarding octave errors) by chance is 3 semitones. Recently, a multi-site study replicated the significance of APM, but also pointed out its weak effect (Frieler et al., 2013). In that study, a meta-analysis on the original study (Levitin, 1994; *n* = 44 for each of two trials) and 6 replication studies (*n* = 250 in total; average *n* = 46.2 ± 2.2 per study) revealed that the hit rates were significantly higher compared to random behavior, but the effect size was much lower in experiments done in 5 labs compared to that in the original study (Levitin, 1994). Statistically, the octave-error corrected deviation from the target is a circular measure (e.g., one semitone up from a deviation of +6 semitones becomes a deviation of –5 semitones). Thus, to test whether the angular mean of signed errors equals to zero (i.e., a null hypothesis assuming uniform distribution around a circle), Rayleigh's test should be used, as done in Frieler et al. (2013). From the published data (Levitin, 1994), we carried out Rayleigh's test (Berens, 2009). The *p*-values were 0.059 and 0.035 for the two songs in the original study (Levitin, 1994) and 0.061 and 0.134 in the pooled data of the replicated study (Frieler et al., 2013). For comparison (although this was not an APM test but an AP test using a digital piano), we also carried out Rayleigh's test on the behavioral data published in Kim and Knösche (2016). The *p*-values were <10^−6^ and 0.438 for musicians with and without AP, respectively, suggesting the accuracy of APM in non-musicians seems to be still far lower compared to that in musicians with AP.

Secondly, related to the first point, it has been implied that so-called “quasi” (or pseudo, latent, implicit)-AP (qAP) musicians might use APM in AP tests. While the operational definition of qAP differs slightly across studies (Bachem, 1937, 1955; Miyazaki, 2004; Athos et al., 2007), it generally refers to an intermediate performance in AP tests (Wilson et al., 2009). In general, highly trained musicians have a very good relative pitch (RP), which is the ability to recognize and manipulate musical intervals and chords in a tonal context. Thus, a highly trained musician who can directly recognize only a few reference tones (i.e., qAP) may perform well above musicians without AP, sometimes even comparably to musicians with AP in terms of accuracy.

Self-descriptions of qAP musicians about their strategies for the AP test reported in a PET study (Wilson et al., 2009) are very insightful although qualitative and subjective in nature. Conditions of confident recognition of pitch chroma were largely different (e.g., specific timbre, octave range, specific pitch chromas). Some qAP musicians reported using a familiar song or musical instrument to form a reference tone. The results indirectly suggest that some musicians with qAP may recall a reference tone, compare it with a given tone, and find the pitch name in relation to the reference in a very short time, presumably facilitated by extensive musical training. This seems to fit better the proposed perceptual subprocess based on APM (Levitin and Rogers, 2005). The question remains whether “true” AP musicians use different mechanisms to directly recognize pitch chroma or a similar but far more efficient mechanism as qAP musicians (Van Hedger et al., 2015a).

Thirdly, APC involves a discrete representation of pitch chroma consistent with standard tuning whereas APM could be misaligned with it. In previously discussed experiments on APM (Levitin, 1994; Frieler et al., 2013), singing performance was analyzed by rounding to the nearest pitch chroma in standard tuning, without reporting the deviations. Thus, these results do not reveal how precise APM in non-AP population is. Conversely, many AP musicians can perceive a slight deviation from standard tuning (0.2–0.4 semitones) and sharply recognize in-tune pitches (Miyazaki, 1988), suggesting that there exists a template of pitch chroma, which is fixed at certain frequencies in musicians with AP.

Very interestingly, however, it has been shown that the pitch chroma template is not as rigid as previously assumed, but can be plastic (Van Hedger et al., 2013). In the experiment, musicians with AP listened to Johannes Brahms's Symphony No. 1 (total 45 min). During the first movement (15 min), the pitch was transposed downwards extremely slowly (0.02 semitones per min) and then kept constant (i.e., 0.33 semitones below standard tuning) for the rest of the piece. After listening to the detuned symphony, musicians with AP made transposed answers, suggesting that the AP template can be (presumably temporarily) affected by the concurrent experience. Another study reported that the precision of AP perception was positively correlated with daily musical experience (Dohn et al., 2014), suggesting that the AP template indeed seems to be refreshed and retuned by daily musical experience. Note that these studies (Van Hedger et al., 2013; Dohn et al., 2014) used a fairly liberal definition of AP (i.e., >68% of a maximum score) according to a large-scale study (Athos et al., 2007). Nonetheless, these studies suggest that when measuring performance level of AP, a participants' recent musical experience should be carefully matched.

## Neural implementation of pitch chroma categorization

As mentioned above, a number of neuroimaging studies reported structural and functional correlates of AP (Schlaug et al., 1995; Keenan et al., 2001; Ohnishi et al., 2001; Itoh et al., 2005; Oechslin et al., 2009; Wilson et al., 2009; Loui et al., 2011; Jäncke et al., 2012; Elmer et al., 2015). However, only a few suggested possible mechanisms for the categorization of pitch chroma.

Previous studies based on manual delineation of the PT point toward a leftward asymmetry of their area/volume, though not because of a larger left PT but a smaller right PT (Schlaug et al., 1995; Keenan et al., 2001; Wilson et al., 2009; Loui et al., 2011). Involvement of the PT in pitch processing has been consistently implicated in a large number of studies (see Griffiths and Warren, 2002 for a review). In particular, parameterized pitch salience was localized in the posterior Heschl's sulcus and anterior PT, suggesting a critical role in pitch extraction (Barker et al., 2012). However, it is currently unknown how pitch extraction is related to pitch chroma extraction (e.g., whether they are carried out separately or simultaneously).

Dohn and colleagues suggested involvement of hippocampal structures based on a correlation between fractional anisotropy (FA) in the right ventral pathway (i.e., the inferior fronto-occipital fasciculus and the inferior longitudinal fasciculus) and cortical thickness in the right parahippocampal gyrus (Dohn et al., 2015). It is commonly known that hippocampal structures are selectively involved in the retrieval of context-based episodic memory but not in familiarity-based recognition (Eldridge et al., 2000; Fortin et al., 2004). In very rare case reports of epileptic patients with AP (Zatorre, 1989; Suriadi et al., 2015), AP recognition in patients was intact after anterior temporal lobectomy of the left hemisphere (Zatorre, 1989) and a selective amygdalohippocampectomy of the right hemisphere (Suriadi et al., 2015). These findings suggest that AP might be relatively independent of medio-temporal structures, particularly the hippocampus.

**Figure 2:**
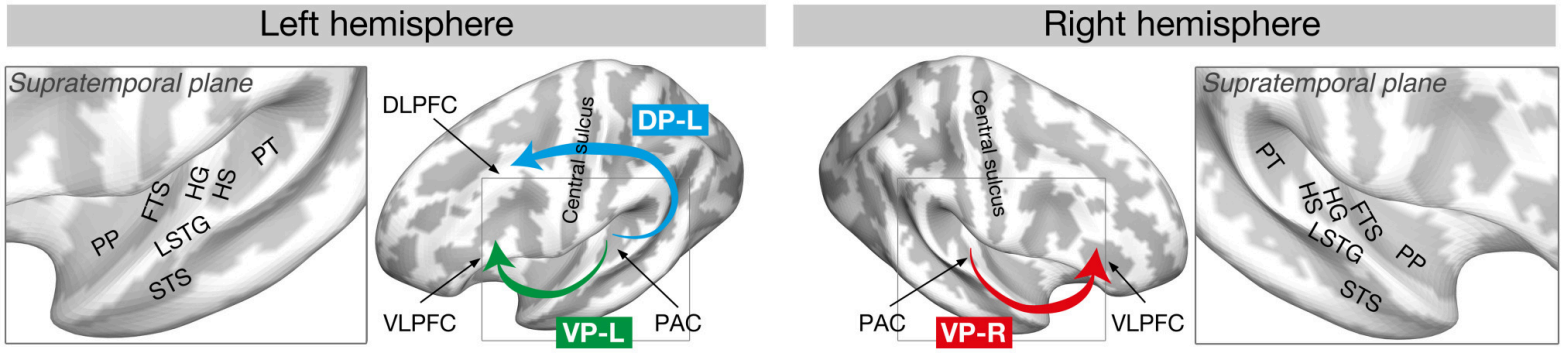
Dorsal and ventral auditory pathways that are found to be relevant to the AP process. Reproduced from Kim and Knösche (2017) with permission by John Wiley and Sons. PP, planum polare; FTS, first transverse sulcus; LSTG, lateral superior temporal gyrus; STS, superior temporal sulcus; HG, Heschl's gyrus; HS, Heschl's sulcus; PT, planum temporale; DLPFC, dorsolateral prefrontal cortex; VLPFC, ventrolateral prefrontal cortex; PAC, primary auditory cortex; DP, dorsal pathway; VP, ventral pathway.

Another suggestion from recent research (Kim and Knösche, 2016, 2017) is based on the dual auditory pathway hypothesis (Rauschecker and Tian, 2000; Rauschecker, 2015). As depicted in Figure 2, the hypothesis suggests that auditory information related to spatial properties (i.e., location or movement) of auditory objects is processed through the dorsal auditory pathway (from the HG to the PT, supramarginal gyrus, and dorsolateral PFC), whereas non-spatial information (i.e., identification and intrinsic characteristics) of auditory objects is processed in the ventral pathway (from the HG to the PP, temporal pole, and ventrolateral PFC) supported by many studies (Kaas and Hackett, 1999; Rauschecker and Tian, 2000; Tian et al., 2001; Warren and Griffiths, 2003; Warren et al., 2003a; Arnott et al., 2004; Kusmierek and Rauschecker, 2009; Rauschecker, 2015).

There is evidence of pitch chroma and pitch height being processed separately in the anterior and posterior parts of the superior temporal planes, respectively (Warren et al., 2003b). It was discussed that the changes in pitch height could be useful for segregating auditory objects (Griffiths and Warren, 2002), whereas changes in pitch chroma can be useful for tracking auditory objects and thus might be related to object identification. In this context, the findings of heavier cortical myelination in the right PP (Kim and Knösche, 2016) and the heightened resting-state functional connectivity of the right PP with the bilateral STSs and left PP in musicians with AP (Kim and Knösche, 2017) could be related to acquisition and preservation of the AP template and its use in pitch chroma extraction.

Also notably, the increase in myelination was found at the middle depth of the cortex (Kim and Knösche, 2016), which suggests enhanced local connectivity amongst neighboring cortical columns in the area. This distinctive connectivity pattern might be one way to implement a system that recognizes a certain pitch chroma from a representation of pitch.

## Open questions and ideas

In this review, we discussed conceptual and neuroscientific issues on the perceptual subprocess of AP. Below, we briefly list a number of interesting open questions and possible ideas regarding answers to them.

1. As reported earlier (Wilson et al., 2009), it appears that some qAPs can directly recognize a limited number of pitch chromas. From those qAPs we may be able to test differences between pitch identification based on APM (for a non-template pitch) and APC (for a template pitch) using a within-subject design experiment.
2. Although the AP template could be affected by recent musical experience, it seems to be able to resolve pitch at high precision (Miyazaki, 1988). Using a tone deviated by 50% semitone from standard tuning might allow one to disentangle physical properties of auditory input and perceived categories in pitch chroma.
3. To address the relationship between pitch extraction and pitch chroma extraction, stimuli used in studies on pitch processing such as iterative-rippled noise (Yost, 1996) can be used to parameterize pitch salience and pitch intonation.

## Author contributions

S-GK and TRK wrote the manuscript together.

### Conflict of interest statement

The authors declare that the research was conducted in the absence of any commercial or financial relationships that could be construed as a potential conflict of interest.

## Footnotes

^1^The notation of pitch (e.g., A4) consists of an alphabetic label of twelve segments within an octave (“pitch chroma”; C, C#, …, A, …) and an integer octave index (“pitch height”; e.g., A3 = 220 Hz, A4 = 440 Hz in standard tuning). The most popular Western musical scale is currently a 12-ton equal temperament (12-TET).

## References

[B1] Arnott S. R. Binns M. A. Grady C. L. Alain C. (2004). Assessing the auditory dual-pathway model in humans. Neuroimage 22, 401–408. 10.1016/j.neuroimage.2004.01.014 15110033

[B2] Athos E. A. Levinson B. Kistler A. Zemansky J. Bostrom A. Freimer N.. (2007). Dichotomy and perceptual distortions in absolute pitch ability. Proc. Natl. Acad. Sci. U.S.A. 104, 14795–14800. 10.1073/pnas.0703868104 17724340 PMC1959403

[B3] Bachem A. (1937). Various types of absolute pitch. J. Acoust. Soc. Am. 9, 146–151. 10.1121/1.1915919

[B4] Bachem A. (1955). Absolute pitch. J. Acoust. Soc. Am. 27, 1180–1185. 10.1121/1.1908155

[B5] Barker D. Plack C. J. Hall D. A. (2012). Reexamining the evidence for a pitch-sensitive region: a human fMRI study using iterated ripple noise. Cereb. Cortex 22, 745–753. 10.1093/cercor/bhr065 21709174

[B6] Ben-Haim M. S. Eitan Z. Chajut E. (2014). Pitch memory and exposure effects. J. Exp. Psychol. Hum. Percept. Perform. 40:24. 10.1037/a0033583 23875573

[B7] Berens P. (2009). CircStat: a MATLAB toolbox for circular statistics. J. Stat. Softw. 31, 1–21. 10.18637/jss.v031.i10

[B8] Bermudez P. Lerch J. P. Evans A. C. Zatorre R. J. (2009). Neuroanatomical correlates of musicianship as revealed by cortical thickness and voxel-based morphometry. Cereb. Cortex 19, 1583–1596. 10.1093/cercor/bhn196 19073623

[B9] Briley P. M. Breakey C. Krumbholz K. (2012). Evidence for pitch chroma mapping in human auditory cortex. Cereb. Cortex 23, 2601–2610. 10.1093/cercor/bhs242 22918980 PMC3792739

[B10] Deutsch D. (2013). The Psychology Of Music. Amsterdam: Academic Press.

[B11] Deutsch D. Henthorn T. (2004). Absolute pitch, speech, and tone language: some experiments and a proposed framework. Music Percept. 21, 339–356. 10.1525/mp.2004.21.3.339

[B12] Dohn A. Garza-Villarreal E. A. Chakravarty M. M. Hansen M. Lerch J. P. Vuust P. (2015). Gray- and white-matter anatomy of absolute pitch possessors. Cereb. Cortex 25, 1379–1388. 10.1093/cercor/bht334 24304583

[B13] Dohn A. Garza-Villarreal E. A. Ribe L. R. Wallentin M. Vuust P. (2014). Musical activity tunes up absolute pitch ability. Music Percept. 31, 359–371. 10.1525/mp.2014.31.4.359

[B14] Eldridge L. L. Knowlton B. J. Furmanski C. S. Bookheimer S. Y. Engel S. A. (2000). Remembering episodes: a selective role for the hippocampus during retrieval. Nat. Neurosci. 3, 1149–1152. 10.1038/80671 11036273

[B15] Elmer S. Rogenmoser L. Kühnis J. Jäncke L. (2015). Bridging the gap between perceptual and cognitive perspectives on absolute pitch. J. Neurosci. 35, 366–371. 10.1523/JNEUROSCI.3009-14.2015 25568128 PMC6605251

[B16] Elmer S. Sollberger S. Meyer M. Jancke L. (2013). An empirical reevaluation of absolute pitch: behavioral and electrophysiological measurements. J. Cogn. Neurosci. 25, 1736–1753. 10.1162/jocn_a_00410 23647515

[B17] Fortin N. J. Wright S. P. Eichenbaum H. (2004). Recollection-like memory retrieval in rats is dependent on the hippocampus. Nature 431, 188–191. 10.1038/nature02853 15356631 PMC4053162

[B18] Frieler K. Fischinger T. Schlemmer K. Lothwesen K. Jakubowski K. Mullensiefen D. (2013). Absolute memory for pitch: a comparative replication of Levitin's 1994 study in six European labs. Music. Sci. 17, 334–349. 10.1177/1029864913493802

[B19] Griffiths T. D. Warren J. D. (2002). The planum temporale as a computational hub. Trends Neurosci. 25, 348–353. 10.1016/S0166-2236(02)02191-4 12079762

[B20] Itoh K. Suwazono S. Arao H. Miyazaki K. Nakada T. (2005). Electrophysiological correlates of absolute pitch and relative pitch. Cereb. Cortex 15, 760–769. 10.1093/cercor/bhh177 15371294

[B21] Jäncke L. Langer N. Hänggi J. (2012). Diminished whole-brain but enhanced peri-sylvian connectivity in absolute pitch musicians. J. Cogn. Neurosci. 24, 1447–1461. 10.1162/jocn_a_00227 22524277

[B22] Kaas J. H. Hackett T. A. (1999). ‘What’ and ‘where’ processing in auditory cortex. Nat. Neurosci. 2, 1045–1047. 10.1038/15967 10570476

[B23] Keenan J. P. Thangaraj V. Halpern A. R. Schlaug G. (2001). Absolute pitch and planum temporale. Neuroimage 14, 1402–1408. 10.1006/nimg.2001.0925 11707095

[B24] Kim S.-G. Knösche T. R. (2016). Intracortical myelination in musicians with absolute pitch: quantitative morphometry using 7-T MRI. Hum. Brain Mapp. 37, 3486–3501. 10.1002/hbm.23254 27160707 PMC5084814

[B25] Kim S.-G. Knösche T. R. (2017). Resting state functional connectivity of the ventral auditory pathway in musicians with absolute pitch. Hum. Brain Mapp. 38, 3899–3916. 10.1002/hbm.23637 28481006 PMC6866733

[B26] Kusmierek P. Rauschecker J. P. (2009). Functional specialization of medial auditory belt cortex in the alert rhesus monkey. J. Neurophysiol. 102, 1606–1622. 10.1152/jn.00167.2009 19571201 PMC2746772

[B27] Levitin D. J. (1994). Absolute memory for musical pitch - evidence from the production of learned melodies. Percept. Psychophys. 56, 414–423. 10.3758/BF03206733 7984397

[B28] Levitin D. J. Rogers S. E. (2005). Absolute pitch: perception, coding, and controversies. Trends Cogn. Sci. 9, 26–33. 10.1016/j.tics.2004.11.007 15639438

[B29] Loui P. Li H. C. Hohmann A. Schlaug G. (2011). Enhanced cortical connectivity in absolute pitch musicians: a model for local hyperconnectivity. J. Cogn. Neurosci. 23, 1015–1026. 10.1162/jocn.2010.21500 20515408 PMC3012137

[B30] Miyazaki K. (1988). Musical pitch identification by absolute pitch possessors. Percept. Psychophys. 44, 501–512. 10.3758/BF03207484 3200669

[B31] Miyazaki K. (2004). How well do we understand absolute pitch? Acoust. Sci. Technol. 25, 426–432. 10.1250/ast.25.426

[B32] Oechslin M. S. Imfeld A. Loenneker T. Meyer M. Jäncke L. (2009). The plasticity of the superior longitudinal fasciculus as a function of musical expertise: a diffusion tensor imaging study. Front. Hum. Neurosci. 3:76. 10.3389/neuro.09.076.2009 20161812 PMC2821183

[B33] Ohnishi T. Matsuda H. Asada T. Aruga M. Hirakata M. Nishikawa M.. (2001). Functional anatomy of musical perception in musicians. Cereb. Cortex 11, 754–760. 10.1093/cercor/11.8.754 11459765

[B34] Rauschecker J. P. (2015). Auditory and visual cortex of primates: a comparison of two sensory systems. Eur. J. Neurosci. 41, 579–585. 10.1111/ejn.12844 25728177 PMC4347938

[B35] Rauschecker J. P. Tian B. (2000). Mechanisms and streams for processing of “what” and “where” in auditory cortex. Proc. Natl. Acad. Sci. U.S.A. 97, 11800–11806. 10.1073/pnas.97.22.11800 11050212 PMC34352

[B36] Schlaug G. Jäncke L. Huang Y. Steinmetz H. (1995). *In vivo* evidence of structural brain asymmetry in musicians. Science 267, 699–701. 10.1126/science.7839149 7839149

[B37] Siegel J. A. (1974). Sensory and verbal coding strategies in subjects with absolute pitch. J. Exp. Psychol. 103, 37–44. 10.1037/h0036844 4423196

[B38] Smith N. A. Schmuckler M. A. (2008). Dial A440 for absolute pitch: absolute pitch memory by non-absolute pitch possessors. J. Acoust. Soc. Am. 123, EL77–EL84. 10.1121/1.2896106 18396925

[B39] Suriadi M. M. Usui K. Tottori T. Terada K. Fujitani S. Umeoka S.. (2015). Preservation of absolute pitch after right amygdalohippocampectomy for a pianist with TLE. Epilepsy Behav. 42, 14–17. 10.1016/j.yebeh.2014.10.025 25499156

[B40] Takeuchi A. H. Hulse S. H. (1993). Absolute Pitch. Psychol. Bull. 113, 345–361. 10.1037/0033-2909.113.2.345 8451339

[B41] Tian B. Reser D. Durham A. Kustov A. Rauschecker J. P. (2001). Functional specialization in rhesus monkey auditory cortex. Science 292, 290–293. 10.1126/science.1058911 11303104

[B42] Van Hedger S. C. Heald S. L. Koch R. Nusbaum H. C. (2015a). Auditory working memory predicts individual differences in absolute pitch learning. Cognition 140, 95–110. 10.1016/j.cognition.2015.03.012 25909580

[B43] Van Hedger S. C. Heald S. L. Nusbaum H. C. (2015b). The effects of acoustic variability on absolute pitch categorization: evidence of contextual tuning. J. Acoust. Soc. Am. 138, 436–446. 10.1121/1.4922952 26233042

[B44] Van Hedger S. C. Heald S. L. Nusbaum H. C. (2013). Absolute pitch may not be so absolute. Psychol. Sci. 24, 1496–1502. 10.1177/0956797612473310 23757308

[B45] Van Hedger S. C. Heald S. L. Nusbaum H. C. (2016). What the [bleep]? Enhanced absolute pitch memory for a 1000Hz sine tone. Cognition 154, 139–150. 10.1016/j.cognition.2016.06.001 27289485

[B46] Ward W. Burns E. (1982). Absolute pitch, in The Psychology of Music, ed Deutsch D. (New York, NY: Academic Press), 265–298.

[B47] Warren J. D. Griffiths T. D. (2003). Distinct mechanisms for processing spatial sequences and pitch sequences in the human auditory brain. J. Neurosci. 23, 5799–5804. 12843284 10.1523/JNEUROSCI.23-13-05799.2003 PMC6741275

[B48] Warren J. D. Uppenkamp S. Patterson R. D. Griffiths T. D. (2003a). Analyzing pitch chroma and pitch height in the human brain. Ann. N.Y. Acad. Sci. 999, 212–214. 10.1196/annals.1284.032 14681144

[B49] Warren J. D. Uppenkamp S. Patterson R. D. Griffiths T. D. (2003b). Separating pitch chroma and pitch height in the human brain. Proc. Natl. Acad. Sci. U.S.A. 100, 10038–10042. 10.1073/pnas.1730682100 12909719 PMC187755

[B50] Wengenroth M. Blatow M. Heinecke A. Reinhardt J. Stippich C. Hofmann E.. (2014). Increased volume and function of right auditory cortex as a marker for absolute pitch. Cereb. Cortex 24, 1127–1137. 10.1093/cercor/bhs391 23302811

[B51] Wilson S. J. Lusher D. Wan C. Y. Dudgeon P. Reutens D. C. (2009). The neurocognitive components of pitch processing: insights from absolute pitch. Cereb. Cortex 19, 724–732. 10.1093/cercor/bhn121 18663250 PMC2638817

[B52] Yost W. A. (1996). Pitch of iterated rippled noise. J. Acoust. Soc. Am. 100, 511–518. 10.1121/1.415873 8675844

[B53] Zatorre R. J. (1989). Intact absolute pitch ability after left temporal lobectomy. Cortex 25, 567–580. 10.1016/S0010-9452(89)80018-8 2612176

[B54] Zatorre R. J. (2003). Absolute pitch: a model for understanding the influence of genes and development on neural and cognitive function. Nat. Neurosci. 6, 692–695. 10.1038/nn1085 12830161

[B55] Zatorre R. J. Perry D. W. Beckett C. A. Westbury C. F. Evans A. C. (1998). Functional anatomy of musical processing in listeners with absolute pitch and relative pitch. Proc. Natl. Acad. Sci. U.S.A. 95, 3172–3177. 10.1073/pnas.95.6.3172 9501235 PMC19714

